# Blood-CNS Barrier Impairment in ALS patients versus an animal model

**DOI:** 10.3389/fncel.2014.00021

**Published:** 2014-02-03

**Authors:** Svitlana Garbuzova-Davis, Paul R. Sanberg

**Affiliations:** ^1^Department of Neurosurgery and Brain Repair, Center of Excellence for Aging and Brain Repair, Morsani College of Medicine, University of South FloridaTampa, FL, USA; ^2^Department of Molecular Pharmacology and Physiology, Morsani College of Medicine, University of South FloridaTampa, FL, USA; ^3^Department of Pathology and Cell Biology, Morsani College of Medicine, University of South FloridaTampa, FL, USA; ^4^Department of Psychiatry, Morsani College of Medicine, University of South FloridaTampa, FL, USA

**Keywords:** amyotrophic lateral sclerosis, blood–CNS barrier, patients, animal models, microvascular pathology

## Abstract

Amyotrophic lateral sclerosis (ALS) is a severe neurodegenerative disease with a complicated and poorly understood pathogenesis. Recently, alterations in the blood–Central Nervous System barrier (B-CNS-B) have been recognized as a key factor possibly aggravating motor neuron damage. The majority of findings on ALS microvascular pathology have been determined in mutant superoxide dismutase (SOD1) rodent models, identifying barrier damage during disease development which might similarly occur in familial ALS patients carrying the SOD1 mutation. However, our knowledge of B-CNS-B competence in sporadic ALS (SALS) has been limited. We recently showed structural and functional impairment in postmortem gray and white matter microvessels of medulla and spinal cord tissue from SALS patients, suggesting pervasive barrier damage. Although numerous signs of barrier impairment (endothelial cell degeneration, capillary leakage, perivascular edema, downregulation of tight junction proteins, and microhemorrhages) are indicated in both mutant SOD1 animal models of ALS and SALS patients, other pathogenic barrier alterations have as yet only been identified in SALS patients. Pericyte degeneration, perivascular collagen IV expansion, and white matter capillary abnormalities in SALS patients are significant barrier related pathologies yet to be noted in ALS SOD1 animal models. In the current review, these important differences in blood–CNS barrier damage between ALS patients and animal models, which may signify altered barrier transport mechanisms, are discussed. Understanding discrepancies in barrier condition between ALS patients and animal models may be crucial for developing effective therapies.

## INTRODUCTION

Amyotrophic lateral sclerosis (ALS) is a neurodegenerative disease affecting upper and lower motor neurons in the brain and spinal cord, damage which leads to progressive muscle atrophy, paralysis and death typically within three to five years from diagnosis (Rowland and Shneider, 2001). Most ALS cases are sporadic amyotrophic lateral sclerosis(SALS) with only 5–10% genetically linked familial amyotrophic lateral sclerosis (FALS); 20% of FALS cases show missense mutations in the Cu/Zn superoxide dismutase (SOD1) gene (Rosen et al., 1993). Clinical presentation and pathology of SALS and FALS, however, are similar. Numerous hypotheses exist regarding ALS pathogenesis (Alexianu et al., 2001; Cleveland and Rothstein, 2001; Bruijn et al., 2004; Strong et al., 2005; Pasinelli and Brown, 2006; Van Den Bosch et al., 2006; Mitchell and Borasio, 2007; Rothstein, 2009; Saleh et al., 2009; Hovden et al., 2013), but the causes of the diffuse motor neuron degeneration are still uncertain.

The blood–Central Nervous System barrier (B-CNS-B) is composed of the blood–brain barrier (BBB), blood–spinal cord barrier (BSCB), and blood–cerebrospinal fluid barrier (BCSFB) and has a crucial role in controlling CNS homeostasis by selective transport of substances to and from the systemic compartment and blocking passive diffusion of harmful blood solutes (Bradbury, 1985; Dermietzel and Krause, 1991; Pardridge, 1991, 1999; Nag, 2003; Vorbrodt and Dobrogowska, 2003; Ballabh et al., 2004). This control is possible due to the unique composition of the microvasculature – capillaries formed by endothelial cells (BBB and BSCB) and epithelial cells of the choroids plexus (BCSFB). Exchange by free diffusion is limited to molecules massing less than 450 Da; more massive substances require specific transport mechanisms. These mechanisms allow influx of required substances and efflux of cell waste (Begley and Brightman, 2003; Begley, 2004; Pardridge, 2005). Endothelial cells and their tight/adherens junctions are the primary components of the BBB and BSCB systems, while other barrier elements (pericytes, astrocytes, perivascular macrophages, and the basal lamina) also have essential roles in the tightly integrated unit maintaining the CNS environment for proper function of neuronal cells. Although major structural and functional properties are shared between the BBB and BSCB, some morphological and physiological differences have been noted in the BSCB (Bartanusz et al., 2011). Glycogen microvessel deposits, increased capillary permeability for some tracers, and decreased tight/adherence junction protein expressions were described for the BSCB in comparison to the BBB. Therefore, dysfunctional or structural impairment of any B-CNS-B component may lead to an increasingly toxic CNS environment. Microvascular endothelial dysfunction, in particular, might be implicated in the pathogenesis of various neurodegenerative diseases (Grammas et al., 2011).

Early studies in the 1980s reported altered BCSFB permeability as indicated by abnormal serum proteins and complement in the CSF of ALS patients (Leonardi et al., 1984; Annunziata and Volpi, 1985). These observations were followed by detection of blood-borne substances in the CNS tissue of ALS patients (Donnenfeld et al., 1984), suggesting BBB and BSCB leakage. Only relatively recent research has focused on microvascular competence in the brain and spinal cord, indicating impairment of BBB and BSCB integrity in animal models of ALS and in ALS patients. Compelling evidence of this B-CNS-B damage has been demonstrated at structural and functional levels in an animal model of ALS at initial stage of disease and this damage was exacerbated with disease progression (Garbuzova-Davis et al., 2007a, b; Zhong et al., 2008; Nicaise et al., 2009a,b; Miyazaki et al., 2011). Importantly, BSCB breakdown was found in SOD1 mutant mice and rats prior to motor neuron degeneration and neuroinflammation (Zhong et al., 2008; Nicaise et al., 2009a; Miyazaki et al., 2011). Evidence of BSCB impairment has also been observed in postmortem tissue from ALS patients. Loss of endothelium integrity, as shown by significant reductions of tight junction proteins and astrocyte end-feet dissociated from the endothelium, was observed in spinal cords from ALS patients (Henkel et al., 2009; Miyazaki et al., 2011). Recently, we showed structural and functional impairment in postmortem gray and white matter microvessels of medulla and spinal cord tissue from SALS patients, suggesting pervasiveness of the B-CNS-B damage (Garbuzova-Davis et al., 2012). These results strengthen the likelihood that barrier disruption contributes to disease pathogenesis (Garbuzova-Davis et al., 2008). However, B-CNS-B disruption could also trigger, as well as aggravate, motor neuron degeneration in ALS. Vascular impairment has only recently been recognized as a key factor in ALS, identifying ALS as a neurovascular disease (Garbuzova-Davis et al., 2011; Rodrigues et al., 2012). Although numerous similarities in barrier damage have been identified in mutant SOD1 animal models of ALS and ALS patients, some differences in the B-CNS-B alterations have been noted. In the current review, these discrepancies in the BBB and BSCB competence between ALS patients and animal models are discussed with an aim toward developing effective new therapies for ALS.

## BBB AND BSCB IMPAIRMENT IN TRANSGENIC RODENT MODELS OF ALS

Only comparatively recent research has focused on investigations of BBB and BSCB integrity in ALS. Initially, Garbuzova-Davis et al. (2007a) demonstrated ultrastructural capillary alterations in the brainstem and spinal cord (cervical and lumbar) in both early and late stages of disease in G93A SOD1 mice. Electron microscopy analysis showed highly vacuolated and degenerated endothelial cells, mitochondrial degeneration within endothelial cells, extensive perivascular edema, and swelling of astrocyte end-feet adjacent to capillaries. Capillary rupture was also indicated by the presence of erythrocytes in the extracellular space of brainstem microvessels in early symptomatic G93A mice. These findings were later confirmed by a study from the same research group (Garbuzova-Davis et al., 2007b), showing Evans blue leakage in spinal cord capillaries of G93A mice at 13 weeks of age, indicating functional impairment of the BSCB in early stage disease. The study also demonstrated endothelial damage through downregulation of the transporter protein Glut-1 and CD146 expressions, associated with decreased laminin, a component of the basement membrane in capillaries. The alterations were mainly detected in the ventral horns of the spinal cords, areas most affected by ALS. Importantly, motor neurons demonstrated intracellular edema and cytoplasmic vacuolization, in addition to degenerated axons with myelin disruption near capillaries in the brainstem and spinal cords of G93A mice at early disease stage.

The study by Zhong et al. (2008) not only confirmed these observations on microvascular barrier damage in the spinal cord of symptomatic G93A mice, but also showed that BSCB disruption precedes neuroinflammation and might initiate disease symptoms. Western blot analysis evidenced diminished levels of zonula occludens-1 (ZO-1), occludin, and claudin-5 tight junction proteins and Glut-1 prior to disease onset in ALS mice. Although these alterations were observed, markers of endothelial activation (intercellular adhesion molecule-1, ICAM-1) and inflammation (monocyte chemoattractant protein-1, MCP-1) and cycloxygenase-2 (COX-2) were not indicated. Also prior to motor neuron loss and inflammatory changes, the investigators showed 10–15% reductions in total capillary length and 30 to 45% decreases in spinal cord blood flow of SOD1 transgenic mice. Additionally, microhemorrhages and hemosiderin deposits were found in spinal cord parenchyma, demonstrating BSCB functional impairment and disruption.

Miyazaki et al. (2011) also evaluated BSCB integrity in G93A SOD1 mice and observed progressive downregulation of occludin and platelet-endothelium cell adhesion molecule-1 (PECAM-1 or CD31) and vascular collagen IV, associated with increased activity of matrix metalloproteinase-9 (MMP-9), indicating endothelial cell and basement membrane involvement in microvascular pathological changes. All of these observations preceded motor neuron death and were in agreement with Zhong et al. (2008). However, the decline of occludin expression was moderate in the spinal cord tissues from ALS mice from 10 to 15 weeks of age and the expression of this protein significantly decreased in late symptomatic mice at 18 weeks of age. Surprisingly, the quantification of collagen IV in gray matter tissue evidenced protein up-regulation, a finding in opposition to immunohistochemical observations in the perivascular areas. These divergent results were ascribed to increased glial production of collagen IV as a consequence of disease progression and neuroinflammation, possibly reflecting an attempt by neural cells to compensate for endothelium disruption. In support of this suggestion, the authors showed higher collagen IV expression in microglia of G93A mice at 18 weeks of age vs. controls. Hence, it is still unclear whether glial cells, especially astrocytes, induce or prevent microvascular CNS barrier damage.

Another study, based on the G93A SOD1 rat model of ALS, demonstrated interesting results. Ultrastructural alterations of the capillaries such as perivascular swollen astrocyte end-feet, Evans blue leakage, reduced mRNA expression of ZO-1 and occludin, and of agrin, a basement membrane component, were observed in animals only at symptomatic stage (Nicaise et al., 2009a). Conversely, IgG and hemosiderin deposits, other indicators of capillary leakage, were detected in the brainstem and lumbar spinal cord of pre-symptomatic ALS rats. Additionally, the same group (Nicaise et al., 2009b) showed increased expression of aquaporin-4 (AQP4) mRNA and protein in the spinal cord gray matter of end-stage SOD1 rats. Immunohistochemistry revealed increased AQP4 in areas surrounding vessels and motoneuron perikaria. Electron microscopic analysis confirmed localization of this protein in association with perivascular swollen astrocytic processes. The authors suggested that the aquaporin channels may promote perivascular edema and AQR4 might be a potential marker of barrier disruption in ALS (Nicaise et al., 2010). Since elevated AQP4 was also detected near motor neurons, it is possible that dysfunctional astrocytes contribute to further motor neuron degeneration. A more recent study by Bataveljić et al. (2012) confirmed overexpression of AQP4 in the brainstem (facial and trigeminal nuclei) and motor cortex of G93A SOD1 rats at end-stage of disease. Notably, increased AQP4 immunoreactivity was observed in astrocytic processes around blood vessels in studied brain areas of ALS rats. In parallel, the authors determined decreased expression of potassium channel (Kir4.1) in the brainstem and cortex of rats by immunolabeling and Western blot analyses. The authors concluded that the functional changes in these channels could reduce astrocytes’ ability to properly maintain water and potassium CNS homeostasis, not only affecting the BBB but also impeding motor neuron survival in ALS. Moreover, increased vascular permeability in the brain was determined in symptomatic G93A SOD1 rats using Gd-DTPA-enhanced MRI (Andjus et al., 2009). In addition to the BBB leakage, the authors showed marked lateral ventricle dilatation in ALS rats as “the most apparent feature of brain tissue atrophy.”

Thus, the compromised BBB and BSCB are evident in the SOD1 animal model of ALS. Endothelial cell degeneration, swollen astrocyte end-feet and dissociation from the endothelium, capillary leakage, perivascular edema, downregulation of tight junction proteins, microhemorrhages, and reduction of basement membrane components are the main hallmarks of the B-CNS-B impairment. This altered vascular barrier, normally preventing entry of various blood-borne harmful substances into the CNS, could contribute to motor neuron death. Although capillary barrier damage in the CNS of both mouse and rat SOD1 models of ALS has been demonstrated prior to motor neuron loss and neuroinflammation, the specific cause of B-CNS-B breakdown has not yet been identified. It is possible that endothelial cells are more susceptible to detrimental involvement of the misfolding mutant SOD1 protein. However, the particular mechanism(s) responsible for the endothelial cell alteration observed in ALS still needs to be determined.

## BBB AND BSCB IMPAIRMENT IN SPORADIC AND FAMILIAL ALS PATIENTS

The transgenic rodent models expressing mutant SOD1 have greatly contributed to the understanding of ALS pathogenesis. Relatively new research on B-CNS-B competence in ALS has largely used mutant SOD1 rodent models, but determination of barrier integrity without involvement of the mutant SOD1 protein is necessary to clarify the pathogenesis of sporadic human ALS cases. ALS is a multifactorial disease with a complexity of underlying intrinsic and extrinsic factors related to motor neuron death. Some of these detrimental factors might be directly associated with B-CNS-B impairment.

Although the B-CNS-B regulates cellular infiltration into the CNS (Engelhardt, 2008), under inflammatory conditions, extensive leukocyte migration into the CNS occurs following cytokine releases from inflammatory/immune cells (de Vries et al., 1997; Sagar et al., 2012; Sallusto et al., 2012). Leukocyte trafficking through the B-CNS-B is a multistep process mediated by adhesion molecules, classified as immunoglobulins, integrins, cadherins, or selectins. These molecules are up-regulated on the surfaces of the endothelial cells, allowing adhesion and migration of cells from the bloodstream to the CNS (Ley et al., 2007) and are often accompanied by an increased influx of serum proteins. In example, Lindsberg et al. (2010) discussed the deleterious role of the mast cell, a potent inflammatory cell, in cerebral ischemia. These cells located within the cerebral microvasculature secrete cytokines, histamine, heparin, and proteases which can degrade the basement membrane and exacerbate barrier damage, promoting edema, prolonged extravasation, and microhemorrhage and attracting new inflammatory cells. The paracellular pathway of cell migration through the capillary wall is most common, but some authors hypothesize that penetration of immune cells into the CNS can also be accomplished through the transcellular pathway with intact tight junctions (Carman and Springer, 2008).

In the CNS tissue of ALS patients, inflammation and immune cell activation have been detected and are associated with motor neuron degeneration (Donnenfeld et al., 1984; Engelhardt and Appel, 1990; Engelhardt et al., 1993, 1995; Henkel et al., 2004; Boillée et al., 2006). Early studies found IgG and C3/C4 complement deposits in the spinal cord and motor cortex tissues from ALS patients (Donnenfeld et al., 1984). Engelhardt and Appel, (1990) also detected active macrophages and IgG within the endoplasmic reticulum of motor neurons in ALS patients. Interestingly, IgG from sera of ALS patients induced death of a motor neuron cell line (VSC 4.1) *in vitro* (Engelhardt et al., 1995). These study results suggest alteration of B-CNS-B permeability and thus recent investigations have begun to focus on potential endothelial barrier damage in ALS patients.

Henkel et al. (2009) demonstrated diminished mRNA expression of occludin and ZO-1 in human lumbar spinal cord tissue from both sporadic and familial forms of ALS. Similarly, decreased immunostaining for occludin was observed in a small cohort of ALS patients (Miyazaki et al., 2011). These results agreed with the experimental findings, confirming loss of endothelial integrity, and indicating BSCB disruption that might contribute to disease pathogenesis.

A study by Garbuzova-Davis et al. (2010) showed a significant reduction in the numbers of circulating endothelial cells in the peripheral blood of ALS patients with moderate or severe disease. Increased circulating endothelial cells is considered a marker for endothelial damage (Blann et al., 2005) and has been noted in several vascular diseases, including acute myocardial infarct and acute ischemic stroke (Nadar et al., 2005; Chong et al., 2006). These unexpected results in ALS may be explained by a lack of endothelial shedding, resulting in the attachment of new endothelial cells over the damaged cells and thus a multilayer endothelium (Garbuzova-Davis et al., 2010). Indeed, electron microscopy images of ALS mouse tissue have revealed multiple layers of endothelial cells in the brain and spinal cord capillaries (Garbuzova-Davis et al., 2007a). Also, a reduction of circulating endothelial cells in peripheral blood of ALS patients with disease progression could be due to impaired re-endothelialization. The structural and functional integrity of the vascular network, normally maintained by continuous renewal of the endothelial cell layer with a low replication rate of 0.1% per day (Hunting et al., 2005), might be weakened in ALS. It is possible that insufficient production of endothelial progenitor cells by the bone marrow might be an issue. Recent reports demonstrated the functional deficiency of bone marrow mesenchymal stromal cell in ALS patients by reductions in pluripotency and secretion of various trophic factors (Koh et al., 2012) as well as by abnormal productions of MMPs and tissue inhibitors of metalloproteinases (TIMPs; Bossolasco et al., 2010).

In our recent study (Garbuzova-Davis et al., 2012), we examined structural and functional integrities of capillaries in the gray and white matter of the brainstem (medulla) and spinal cord (cervical and lumbar) in postmortem tissue from SALS patients. Study results showed capillary ultrastructural abnormalities in CNS tissues from SALS patients, similar to results from our animal studies (Garbuzova-Davis et al., 2007a). Mainly, severe intra- and extracellular edema, endothelial cell impairment as characterized by swelling and cytoplasmic vacuolization, pericyte degeneration, and degeneration of astrocyte end-feet processes surrounding capillaries were determined by electron microscopic analysis of the medulla and spinal cords. Also, separation of the endothelial cells from the basement membrane, allowing plasma to contact the basal lamina, was a significant capillary alteration noted in brain and spinal cord tissues of SALS patients. Observed capillary endothelium damage led to vascular leakage in the brain and spinal cord as determined by immunostaining for endogenous IgG, confirming previous study results on an animal model of ALS (Garbuzova-Davis et al., 2007b; Nicaise et al., 2009a). Microvascular leakage was also determined in CNS tissues from SALS patients by perivascular fibrin deposits in our electron microscopy images and was recently confirmed (Winkler et al., 2013). Winkler et al. (2013) additionally demonstrated parenchymal accumulation of the plasma-derived proteins trombin and IgG as well as erythrocyte-derived hemoglobin and iron-containing hemosiderin in the cervical gray matter from both SALS and FALS patients via immunostaining. The authors noted that these abnormal deposits in the postmortem tissues were detected only in tissues from ALS patients, but not from controls, and there were unusually widespread pathological depositions at a significant distance from capillaries. In our study (Garbuzova-Davis et al., 2012), fibrin filament deposits and IgG leakage, determined by electron microscopic and immunostaining analysis respectively, were predominantly limited to within the capillary basement membrane or were in close proximity to capillaries. Moreover, erythrocyte extravasation was not determined perivascularly or at neuropil locations in our numerous electron microscope images at different magnifications of the medulla, cervical and lumbar spinal cords from ALS patients. Microhemorrhages within CNS tissues in ALS are possible, even though not supported by MRI evaluations of microbleeds in the brain of ALS patients (Verstraete et al., 2010), so the presence of erythrocytes in CNS parenchyma observed by Winkler et al. (2013) needs further confirmation. Erythrocytes are normally restricted from entry into the CNS and these cells typically extravasate into CNS tissue due to capillary rupture as shown in multiple sclerosis (Adams, 1988), post-traumatic epilepsy (Willmore and Triggs, 1984), and cerebral ischemia (Simard et al., 2007). However, capillary rupture was not evident morphologically in the brain or spinal cords from ALS patients, even by electron microscopy imaging.

Importantly, complementary approaches have identified pericyte degeneration by electron microscopy (Garbuzova-Davis et al., 2012) and shown reduction in pericyte numbers via immunostaining (Winkler et al., 2013) in capillaries of ALS patients, deficiencies which might severely compromise the B-CNS-B. Hence, the cause(s) of pericyte deterioration in human ALS tissues should be determined.

Additionally, analysis of tight junction protein expressions in our study (Garbuzova-Davis et al., 2012) using Western immunoblot showed significant decreases of primarily ZO-1 expression in gray and white matter in all examined SALS tissues, similarly to previous studies (Henkel et al., 2009; Miyazaki et al., 2011). For occludin and claudin-5, diminished protein expressions were mostly found in ALS medulla and cervical spinal cord.

Moreover, our new findings (Garbuzova-Davis et al., 2012) showed extensive vascular basement membrane collagen IV accumulation, 2–2.5 times higher than controls, in the majority of brain and spinal cord vessels from SALS patients. Also, collagen fiber calcifications were determined in some CNS capillaries. Although similar results were noted in Alzheimer’s patients (Claudio, 1996), some previous reports conflict with our study results. Decreased perivascular collagen IV was noted in postmortem ALS spinal cord tissue (Ono et al., 1998; Miyazaki et al., 2011). This discrepancy needs clarification.

Finally, our study results showed a significant increase of microvascular density in gray matter of the lumbar spinal cord from SALS patients vs. controls (Garbuzova-Davis et al., 2012) suggesting that neovascularization occurred to compensate for vascular insufficiency due to dysfunctional capillaries. Currently, we are investigating possible new vessel formation in both an ALS mouse model and ALS patients. Supporting the likelihood of ALS neovascularization, Biron et al. (2011) similarly (to our results) demonstrated increased microvascular density in brains from Alzheimer’s patients, suggesting a relationship between hypervascularity, neoangiogenesis and BBB disruption. Also, ongoing angiogenesis resulted in increased vascular density in postmortem brain tissues, mainly in the hippocampus, in Alzheimer’s patients (Desai et al., 2009). Recently, Desai Bradaric et al. (2012) showed the presence of newly created vessels in postmortem brain tissues such as the substantia nigra pars compacta, locus ceruleus, and putamen from subjects with Parkinson’s disease and progressive supranuclear palsy. The authors suggest that these new angiogenic vessels could contribute to disease inflammatory processes by failing to restrict extravasation of immune cells and inflammatory or toxic factors from the peripheral circulation to the CNS due to the new vessels having incompletely developed BBB properties. This possibility might also be an issue for ALS.

Thus, there is compelling evidence of BBB and BSCB impairment in areas of motor neuron degeneration in ALS patients. Importantly, microvascular alterations seen in both gray and white matter of medulla, cervical, and lumbar spinal cord from SALS patients indicate pervasive B-CNS-B damage that might contribute to disease pathogenesis.

## SIMILARITIES AND DIFFERENCES IN THE B-CNS-B IMPAIRMENT BETWEEN ALS PATIENTS AND ANIMAL MODELS OF ALS

Convincing findings indicate B-CNS-B alterations in both ALS patients and the SOD1 animal model of ALS and suggest these alterations as a possible factor aggravating motor neuron damage. Numerous signs of barrier damage, such as endothelial cell degeneration, capillary leakage, perivascular edema, downregulation of tight junction proteins, and microhemorrhages are common in both mutant SOD1 animal models of disease and ALS patients. To date, other pathogenic features linked to barrier alterations have so far only been identified in ALS patients. Mainly, pericyte degeneration, perivascular basement membrane collagen IV expansion, and white matter capillary abnormalities in SALS patients are significant barrier related pathologies yet to be noted in ALS SOD1 animal models. In **Table 1**, current evidence of B-CNS-B impairment in ALS is provided from animal and human studies.

**Table 1:** Evidence of blood–CNS barrier impairment in ALS patients and SOD1 animal models of ALS.

Description of evidence	References	
	Animal model of ALS	Human ALS
Endothelial cell degeneration or damage	Garbuzova-Davis et al. (2007a), Nicaise et al. (2009a), Miyazaki et al. (2011)	Garbuzova-Davis et al. (2012)
Capillary leakage	Garbuzova-Davis et al. (2007b), Nicaise et al. (2009a), Andjus et al. (2009)	Garbuzova-Davis et al. (2012), Winkler et al. (2013)
Pericyte degeneration or damage		Garbuzova-Davis et al. (2012), Winkler et al. (2013)
Perivascular edema	Garbuzova-Davis et al. (2007a)	Garbuzova-Davis et al. (2012)
Astrocyte end-feet capillary damage and dissociation	Garbuzova-Davis et al. (2007a,b), Nicaise et al. (2009a), Miyazaki et al. (2011), Bataveljić et al. (2012)	Miyazaki et al. (2011), Garbuzova-Davis et al. (2012)
Altered basement membrane components	Garbuzova-Davis et al. (2007b), Nicaise et al. (2009a), Miyazaki et al. (2011)	Ono et al. (1998), Miyazaki et al. (2011), Garbuzova-Davis et al. (2012)
Microhemorrhages or perivascular hemosiderin	Garbuzova-Davis et al. (2007a), Zhong et al. (2008), Nicaise et al. (2009a)	Verstraete et al. (2010) (unsupporting), Winkler et al. (2013)
Altered blood flow or capillary lengths and diameters	Zhong et al. (2008), Miyazaki et al. (2011)	Rule et al. (2010)
Downregulation of junctional complex proteins	Zhong et al. (2008), Nicaise et al. (2009a), Miyazaki et al. (2011)	Henkel et al. (2009), Miyazaki et al. (2011), Garbuzova-Davis et al. (2012)
Altered endothelial transporter protein expression	Garbuzova-Davis et al. (2007b), Zhong et al. (2008), Milane et al. (2010), Jablonski et al. (2012)	Jablonski et al. (2012)

Severe capillary pericyte damage (Garbuzova-Davis et al., 2012; Winkler et al., 2013) is an important finding in ALS patients. At the ultrastructural level, complete pericyte degeneration or cell fragmentation in the adjacent extracellular space was determined in numbers of gray and white matter microvessels in the medulla and cervical/lumbar spinal cord of SALS patients (Garbuzova-Davis et al., 2012). Pericytes play essential roles in maintaining B-CNS-B integrity by regulating capillary permeability, blood flow, vascular tone, and angiogenesis (Hirschi and D’Amore, 1996; Kutcher and Herman, 2009; Armulik et al., 2010; Winkler et al., 2011, 2012; Sá-Pereira et al., 2012). These functions are associated with the cells’ anatomical location, in close proximity to endothelial cells and sharing a common basement membrane. Pericytes are also involved in modulation of immunological response by their phagocytic function (Balabanov and Dore-Duffy, 1998; Dalkara et al., 2011). For example, amyloid deposits within degenerating pericytes were detected in the brains of Alzheimer’s patients (Dalkara et al., 2011). The authors discussed the role of pericyte dysfunction in cerebral hypoperfusion and suggest that “microvascular dysfunction due to pericyte degeneration initiates secondary neurodegenerative changes” (Dalkara et al., 2011). In ALS, Rule et al. (2010) reported reduced capillary blood flow in brains of patients correlating with disease severity, a reduction likely associated with pericyte impairment. A similar decrease in blood flow was determined in the spinal cord of G93A SOD1 mice preceding inflammation and motor neuron injury (Zhong et al., 2008). However, there were no obvious abnormalities detected in pericyte morphology in the brainstem or spinal cord capillaries via electron microscope even in late symptomatic G93A mice (Garbuzova-Davis et al., 2007a). This discrepancy between animal data and results from ALS patients should be investigated.

Another difference between human and animal studies in ALS is vascular basement membrane collagen IV abnormalities. As we noted above, our recent study (Garbuzova-Davis et al., 2012) showed extensive basement membrane collagen IV accumulation and even collagen fiber calcifications in numerous capillaries in gray and white matter brain and spinal cords from SALS patients. However, opposing reports demonstrated decreased perivascular collagen IV in postmortem ALS spinal cord tissue (Ono et al., 1998; Miyazaki et al., 2011). In a study by Miyazaki et al. (2011), reduction of immunoexpression for collagen IV was observed in the anterior horns of the spinal cord from ALS patients as well as in G93A mice during disease progression and was accompanied by MMP-9 up-regulation. The authors conclude that this vascular damage is common to humans and this ALS animal model. However, the observed collagen IV reduction might be due to the diminished capillary density described in the same study. In addition, the authors provided conflicting data with double immunofluorescence staining for collagen IV and Iba-1 (microglial marker) in the spinal anterior horn of G93A mice at 18 weeks of age showing higher collagen IV expression vs. controls along with the appearance of collagen IV-positive microglia. Microglial cells are resident cells in the CNS with macrophagic properties upon their activation and likely overexpression of collagen IV by microglia indicates uptake of this protein. Supportive evidence of the ability of microglia to express collagen IV was not provided (Miyazaki et al., 2011). Also, it is unclear if this collagen IV immunostaining was also associated with capillaries or astrocytes. Double immunostaining for collagen IV and astrocytes might be a reasonable confirming procedure. It has been shown that reactive astrocytes expressed type IV collagen after spinal cord injury in promoting glial scar formation (Liesi and Kauppila, 2002). In the same study, the authors reported that IL-1 beta and TGF beta-1 cytokines induced collagen IV expression in astrocytes *in vitro*. Since reactive astrogliosis and microglia activation are major contributors to inflammatory processes in ALS by secretion of various pro-inflammatory cytokines, particularly TNF-alpha and TGF beta-1 (Schiffer et al., 1996; Hall et al., 1998; McGeer and McGeer, 1998; Elliott, 2001; McGeer and McGeer, 2002; Consilvio et al., 2004; Henkel et al., 2004; Xie et al., 2004; Vargas and Johnson, 2010; Appel et al., 2011; Haidet-Phillips et al., 2011; Sica, 2012; Evans et al., 2013; Phatnani et al., 2013) it is possible that reactive astrocytes not only affect motor neurons but also promote a collagenous vascular basement membrane. Moreover, inflammation may initiate barrier damage by impairing endothelial cell function. Mantovani et al. (1992) showed that the inflammatory environment in ALS affected endothelial cell gene expression, altering cell function. An additional discrepancy between our and the above mentioned studies regarding basement membrane collagen deposition was discussed in detail (Garbuzova-Davis et al., 2012) and primarily focused on the potential imbalance between MMPs and TIMPs due to defective regulation of the MMP pathway by damaged endothelial cells. Also, since downregulation of other basement membrane components such as laminin (Garbuzova-Davis et al., 2007b) and argin (Nicaise et al., 2009a) has been shown in symptomatic G93A mice but not yet confirmed in ALS patients, it is possible that buildup of perivascular collagen IV occurs as a compensatory mechanism for maintenance of vascular integrity.

We strongly believe that abnormal perivascular collagen accumulation in SALS patients takes place over a long period of time. Our notion is partially supported by Ono et al. (1998) who showed widely separated and fragmented collagen bundles in the interstitial tissue surrounding capillaries in the posterior half of lateral funiculus and anterior horn of cervical spinal cord from ALS patients by ultrastructural analysis. Yet total collagen content determined for each of these spinal cord regions was lower in ALS patients than in controls with or without neurological diseases. Although these data are interesting, the existence of basement collagen abnormalities needs clarification.

Thus, the complexity of the B-CNS-B alterations in ALS is evident. Commonalities in barrier pathologies between humans and an animal model of ALS are essential for understanding involvement of the B-CNS-B in disease pathogenesis. However, the disparities in barrier competence in humans with ALS vs. animal model should be considered. Despite a growing research effort, more studies are needed to reveal specific mechanisms of barrier breakdown in ALS. The question still remains: is barrier damage an initial disease factor or a secondary element in human ALS?

## CONCLUSION

ALS has been, and remains, a challenge for developing therapeutics. More than 30 drug compounds have already been tested in ALS clinical trials yet the only modestly effective treatment is riluzole (Aggarwal and Cudkowicz, 2008). Some tested substances failed to prove effective and even showed harmful effects in Phase III clinical trials: IGF-1 (insulin-like growth factor type 1; Sorenson et al., 2008), minocycline (Gordon et al., 2007), creatine (Groeneveld et al., 2003), and topiramate (Cudkowicz et al., 2003).

These disappointing results might reflect defective transport systems in damaged BBB and BSCB in ALS. Degeneration of endothelial and pericyte cells, compromising vascular barrier integrity in the brain and spinal cord in ALS patients, could be the main obstacles for effective drug delivery to the CNS. Also, it is hard to imagine proper transport of pharmaco-therapeutics across an extensively expanded vascular collagenous basement membrane. Such abnormal buildup of basement membrane collagen seems likely to alter barrier influx and efflux transport systems and, as result, motor neurons might suffer both from reduced nutritional deliveries and increased metabolite levels. Additionally, increases of P-glycoprotein (P-gp) along with the breast cancer resistance protein (BCRP) were determined in brain and spinal cord microvessels in both SOD1 animal models and ALS patients (Milane et al., 2010; Jablonski et al., 2012). Jablonski et al. (2012) conclude that the impairment of these P-gp and BCRP efflux transporters might induce pharmaco-resistance in ALS.

Numerous comprehensive reviews discuss therapeutic strategies of transport drugs across the blood–CNS barrier (Abbott and Romero, 1996; Pardridge, 2002; Misra et al., 2003; McCarty, 2005; Patel et al., 2009; Pathan et al., 2009; Gabathuler, 2010) including chemical (i.g., lipid-mediated transport), biological (i.g., specific transporters for pharmaceuticals), or particular drug carrier systems. Also, various drug delivery systems (e.g., liposomes, nanoparticles, or microspheres) and routes (e.g., intranasal, intraventricular, or intrathecal) have been proposed. However, all of these various strategies for effective drug delivery to the CNS rely on a normally functioning BBB/BSCB. In ALS, deliveries of therapeutic drugs are likely to be complicated by the pervasiveness of the B-CNS-B damage.

In conclusion, the blood–CNS barrier should be considered as a primary therapeutic target prior to development of any treatment approach for ALS.

## Conflict of Interest Statement

The authors declare that the research was conducted in the absence of any commercial or financial relationships that could be construed as a potential conflict of interest.
